# Association of low-level blood lead with serum uric acid in U.S. adolescents: a cross-sectional study

**DOI:** 10.1186/s12940-019-0524-0

**Published:** 2019-10-16

**Authors:** Guiping Hu, Guang Jia, Shichuan Tang, Pai Zheng, Lihua Hu

**Affiliations:** 1 School of Medicine, Beihang University, Beijing, 100191 China; 2 Beijing Advanced Innovation Center for Big Data-Based Precision Medicine, Beihang University, Beijing, 100191 China; 3 Beijing Advanced Innovation Center for Biomedical Engineering, Beihang University, Beijing, 100083 China; 4 Department of Occupational and Environmental Health Sciences, School of Public Health, Peking University, Beijing, 100191 China; 5 Key Laboratory of Occupational Safety and Health, Beijing Municipal Institute of Labor Protection, Beijing, 100054 China; 6 Department of Cardiovascular Medicine, the Second Affiliated Hospital of Nanchang University, No. 1 Minde Road, Nanchang, 330006 Jiangxi China

**Keywords:** Blood lead levels, Serum uric acid, Adolescents, NHANES

## Abstract

**Background:**

Uncertainty remains regarding the association between blood lead levels (BLL) and serum uric acid (SUA) with relatively low BLL exposure because of limited data in the adolescent population. We examined the association between BLL and SUA in U.S. adolescents.

**Methods:**

In this cross-sectional study, 8303 adolescents aged 12–19 years from NHANES 1999–2006 were analyzed. BLL was Ln-transformed for analysis for the skewed distribution. Elevated SUA was defined as ≥5.5 mg/dL. Multivariate linear and multiple logistic regression analyses were performed to evaluate the association of BLL with SUA and elevated SUA. Moreover, a generalized additive model (GAM) and a fitted smoothing curve (penalized spline method) were conducted.

**Results:**

The overall mean BLL was 1.3 μg/dL. Multivariate linear regression analyses showed that LnBLL was independently and positively correlated with SUA level (β = 0.13, 95%CI: 0.09–0.17). Multiple logistic analyses showed that LnBLL was associated with a 24% increased prevalence of elevated SUA (OR = 1.24; 95% CI, 1.11–1.38). Analyses using restricted cubic spline confirmed that the associations of LnBLL with SUA and elevated SUA were linear. Subgroup analyses showed that stronger associations between LnBLL and SUA were detected in adolescents with lower levels of education and estimated glomerular filtration rate (eGFR) (all *P* for interaction < 0.05).

**Conclusions:**

BLL was independently and positively correlated with SUA level and elevated SUA among U.S. adolescents, particularly with lower levels of education and eGFR. The data suggest that there is no “safe” threshold level of exposure to lead.

**Graphical abstract:**

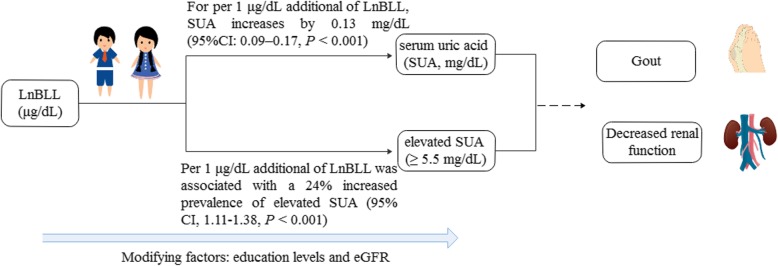

## Introduction

Lead, a heavy metal, is widely distributed in the environment and remains a major public health challenge [1]. In recent years, lead exposure has declined sharply, particularly in developed countries, but chronic low-dose exposures are still a major public health concern [2, 3]. Lead battery workers are primarily exposed to lead in their places of employment, while the general population are exposed to lead from other sources, such as the drinking water supply [4] and air pollution [5]. Early-life exposure to lead, even at low levels, has been associated with a range of adverse health outcomes, including poor growth in childhood [6], impaired cognitive and behavioral functions [7, 8], and hematopoietic system toxicity [9]. In 1991, the Centers for Disease Control and Prevention (CDC) of the United States advised that blood lead levels (BLL) < 10 μg/dL was the safe range for children. However, subsequently, the CDC emphasized that there was no “safe” threshold for BLL in young children because of its established toxicity even at low levels.

Previous epidemiological studies in adults have reported that BLL exposure was positively associated with serum uric acid (SUA) level among lead-exposed workers [10–12]. Association between lead and SUA has been examined in populations encompassing a wide range of lead doses. Despite excessive tubular reabsorption [13] and extrarenal mechanisms of porphyrin metabolism [14], lead exposure causing urate tubular secretion defects is considered to be a major factor involved [15]. As SUA is the final oxidation product of purine metabolism, it has been widely used for evaluating hyperuricemia and gout. An increasing body of epidemiologic evidence shows that elevated SUA or hyperuricemia is associated with a wide variety of adverse health outcomes, including gout, hypertension, obesity, dyslipidemia, diabetes, cardiovascular disease, intellectual disabilities and all-cause mortality [16–20]. However, the association between BLL at a very low level and SUA among adolescents remains unclear because of limited data. In addition, elevated SUA is increasingly prevalent among children and adolescents in the United States [16]. Thus, it is important for researchers to study and understand the association between SUA and continuous lead exposure from the everyday environment.

In the present study, we examined the association between BLL and SUA in a representative sample of US adolescents with relatively low BLL exposure from the National Health and Nutrition Examination Survey (NHANES) from 1999 to 2006.

## Methods

### Study design and population

National Health and Nutrition Examination Survey (NHANES), conducted by the Centers for Disease Control and Prevention (CDC), was an ongoing repeated cross-sectional study designed to assess the lifestyle, health, and nutrition status of non-institutionalized civilian US population through complex, multistage probability sampling [21, 22]. NHANES study protocols were approved by the research ethics review board of the National Center for Health Statistics. Written informed consent was acquired from each participant and obtained from the guardians of participants younger than 18 years of age, and assent was obtained from those aged 12 to 17 years. More detailed information is available at http://www.cdc.gov/nchs/nhanes/nhanes_questionnairees.htm. The NHANES datasets are available on DataDryad (10.5061/dryad.d5h62).

This study used data from the 1999 to 2006 NHANES. Fasting blood samples were collected from those 12 years of age and older. In total, 9493 eligible adolescents aged 12–19 years were enrolled. We excluded participants with missing SUA value (*n* = 1184) and BLL value (*n* = 6).

### Exposure variable and outcomes

In our study, the exposure variable was BLL. BLL was measured at the CDC’s National Center for Environmental Health, Division of Laboratory Sciences using a Perkin-Elmer model SIMAA 6000 simultaneous multielement atomic absorption spectrometer with Zeeman background correction. The analytical laboratory followed extensive quality control procedures. National Institute of Standards and Technology Standard Reference Materials whole-blood materials were used for external calibration. The limit of detection (LOD) for blood lead was 0.3 μg/dL in NHANES from 1999 to 2004. The LOD for blood lead was 0.25 μg/dL in NHANES from 2003 to 2006. Lead concentration below the level of detection was assigned the limit of detection divided by the square root of 2, as recommended by NHANES. The interassay coefficients of variation ranged from 3.1 to 4.0% for BLL. Details of these assays and quality standards are available at http://cdc.gov/nchs/nhanes.

The outcome variable was SUA. SUA was measured on a Roche Hitachi Model 917 or 704 Multichannel Analyzer in 1999–2001 and a Beckman Synchron LX20 in 2002 using a colorimetric method. Serum creatinine level was measured with the same instruments using the Jaffe kinetic alkaline picrate method. Serum creatinine was corrected to standardize to a “gold” standard reference method as recommended by NHANES. The distribution of creatinine and uric acid results from the two laboratories were compared at the time of transition, and no significant differences were observed.

Estimated glomerular filtration rate (eGFR, milliliters per minute per 1.73 m^2^) was calculated via the creatinine-based formula of Schwartz: eGFR = k (height in centimeters)/(serum creatinine in milligrams per deciliter), where k is 0.7 in boys and 0.55 in girls [23, 24].

### Covariates

We selected the appropriate covariates based on previous studies examining risk factors for SUA as well as adjusting for covariates that, when added to this model, changed the matched odds ratio by at least 10% [25, 26]. Therefore, the following variables were used to construct the fully adjusted model: continuous variables included age (years), body mass index (BMI, kg/m^2^), blood pressure (BP, mmHg), poverty to income ratio, dietary data [calcium (mg), total monounsaturated fatty acids (TMFA, gm), total polyunsaturated fatty acids (TPFA, gm), total saturated fatty acids (TSFA, gm), total fat (gm), protein (gm)] and laboratory data [blood cadmium (ug/L), serum cotinine (ng/mL), hemoglobin (g/dL), fasting blood glucose (FBG, mg/dL), total cholesterol (TC, mg/dL), triglycerides (mg/dL), high density lipoprotein cholesterol (HDL-C, mg/dL), blood urea nitrogen (BUN, mg/dL), C-reactive protein (CRP, mg/dL)]; categorical variables consisted of race/ethnicity (non-Hispanic White, non-Hispanic Black, Mexican American, other Hispanic or other), education (less than high school, high school/equivalent, or greater than high school), physical activity (sedentary, low, moderate, high).

### Statistical analysis

The statistical analyses were performed according to the guidelines of the CDC (https://wwwn.cdc.gov/nchs/nhanes/tutorials/default.aspx). Sample weights were used for analyses to account for the complex survey design and non-response of NHANES. We calculated the sample weight for the 8 years of data from 1999 to 2006 as WT_99–06_ = (1/2) × WT_99–02_ + (1/4) × WT_03–04_ + (1/4) × WT_05–06_, where WT_99–02_ is the variable WTMEC4YR from the NHANES 1999–2000 and NHANES 2001–2002; WT_03–04_ and WT_05–06_ were the variable WTMEC2YR from the NHANES 2003–2004 and NHANES 2005–2006 demographic file, respectively [27].

The major question of interest was whether BLL would be associated with SUA after the data was controlled for confounders which might affect SUA. We first examined the distribution of BLL according to the population characteristics listed above. Weighted means, proportions and standard error (Se) were calculated for baseline characteristics using survey sample weights. Because the distribution of values for BLL was strongly skewed toward the upper end, the BLL was Ln-transformed for analysis. Second, we applied multivariate linear regression analysis to evaluate the independent association between LnBLL and SUA. We used four levels of adjustment: Model 1 was adjusted for sociodemographic variables; model 2 was further adjusted for blood biochemical examinations; model 3 was additionally adjusted for dietary data. When model 4 was adjusted for sex, age, BMI, race, hemoglobin, HDL-C and eGFR covariates, the matched odds ratio changed by at least 10%. Third, although there is no universally definition of hyperuricemia in adolescents, previous studies reported that a SUA ≥ 5.5 mg/dL was associated with the risk of hypertenison [16, 28]. Based on this association, the elevated SUA was defined as ≥5.5 mg/dL. We investigated the association of LnBLL with elevated SUA using multivariate binary logistic regression analysis.

The subgroup analyses were performed using stratified multivariate regression analyses. To further characterize the shape of the relationship between BLL and SUA, we used a generalized additive model (GAM) and a fitted smoothing curve (penalized spline method). To ensure the robustness of data analysis, we also did the sensitivity analyses. We converted the LnBLL into a categorical variable, and calculated the *P* for trend. The purpose was to verify the results of LnBLL as the continuous variable and to observe the possibility of nonlinearity.

All the analyses were performed using the statistical package R (http://www.R-project.org, The R Foundation) and Empower (R) (www.empowerstats.com; X&Y Solutions, Inc., Boston, MA). A two-sided *P*-value < 0.05 was considered statistically significant.

## Results

Based on the inclusion and exclusion criteria, a total of 8303 study participants aged 12–19 years (mean age: 15.5 ± 2.3 years; 50.4% men;) from NHANES 1999–2006 were included in this final data analysis (1999–2002: 2106 subjects; 2001–2002: 2192 subjects; 2003–2004: 2036 subjects; 2005–2006: 1969 subjects). Overall, the mean SUA level was 5.0 mg/dL, and 33.2% had a SUA level ≥ 5.5 mg/dL. The overall mean BLL was 1.3 μg/dL. The weighted distributions of selective participants sociodemographic characteristics and other covariates according to LnBLL quartiles are presented in Table 1. The ranges of LnBLL quartiles 1–4 were < − 0.22, − 0.22-0.18, 0.18–0.64 and ≥ 0.64 μg/dL, respectively. There were significant differences between LnBLL quartiles, except for SBP, TPFA and TSFA. Participants with the highest LnBLL in Q4 (BLL ≥ 1.9 μg/dL) were more likely to be boys, to be non-Hispanic black and Mexican American, to have lower educational levels and physical activity, to be of younger age, to have lower values in BMI, DBP, calcium intake, TMFA intake, TC, triglycerides, HDL-C and CRP, and to have higher values in blood cadmium, serum cotinine, hemoglobin, FBG, SUA, eGFR and BUN than those of the other groups (all *P* <  0.05).

**Table 1:** Weighted characteristics of study population based on LnBLL quartiles

Variables^*^	LnBLL quartiles^†^, μg/dL				
	Q1 (< −0.22)	Q2 (−0.22–0.18)	Q3 (0.18–0.64)	Q4 (≥ 0.64)	*P* value
N	2584	2447	1918	1354	
Male, %	34.08 (1.29)	51.28 (1.57)	66.88 (1.65)	77.39 (1.83)	< 0.001
Age, years	15.63 (0.08)	15.52 (0.08)	15.30 (0.08)	15.18 (0.12)	< 0.001
BMI, kg/m^2§^	23.86 (0.18)	23.50 (0.18)	22.99 (0.19)	22.71 (0.22)	< 0.001
SBP, mm Hg	108.98 (0.44)	109.62 (0.29)	110.06 (0.34)	109.92 (0.58)	0.062
DBP, mm Hg	61.57 (0.47)	61.69 (0.40)	60.72 (0.63)	59.74 (0.55)	0.029
Race, %					< 0.001
Non-Hispanic White	69.94 (1.79)	63.49 (2.13)	53.16 (2.3)	42.45 (3.48)	
Non-Hispanic Black	10.07 (1.34)	13.92 (1.22)	19.41 (1.71)	23.11 (2.63)	
Mexican American	9.78 (0.99)	9.87 (1.04)	12.60 (1.54)	18.35 (2.31)	
Other Hispanic	5.26 (0.79)	6.25 (1.04)	6.79 (1.24)	8.68 (1.69)	
Other race	4.95 (0.75)	6.47 (0.91)	8.04 (1.51)	7.41 (2.29)	
Education, %					< 0.001
< high school	82.09 (1.40)	81.35 (1.54)	85.28 (1.5)	87.23 (1.54)	
High school	8.8 (0.88)	9.53 (1.15)	9.00 (1.33)	5.84 (0.90)	
> high school	9.11 (0.99)	9.12 (0.92)	5.72 (0.85)	6.93 (1.13)	
Physical Activity, %^¶^					< 0.001
Sedentary	10.6 (1.03)	10.87 (1.26)	12.31 (2.04)	21.03 (3.02)	
Low	25.47 (1.84)	23.96 (1.79)	21.5 (2.44)	19.15 (2.45)	
Moderate	20.73 (1.27)	14.67 (1.35)	18.85 (2.36)	14.38 (2.26)	
High	43.2 (1.87)	50.49 (1.67)	47.33 (2.64)	45.45 (3.42)	
Dietary					
Calcium, mg	1034.90 (20.30)	983.62 (18.67)	1014.37 (27.42)	927.54 (29.86)	0.032
TMFA, gm	30.44 (0.46)	32.1 (0.49)	32.67 (0.57)	31.99 (0.83)	0.024
TPFA, gm	16.08 (0.29)	16.29 (0.33)	16.94 (0.34)	16.49 (0.46)	0.175
TSFA, gm	28.41 (0.45)	29.72 (0.46)	30.43 (0.6)	29.48 (0.86)	0.058
Total fat, gm	81.35 (1.22)	84.92 (1.23)	87.02 (1.52)	84.88 (2.19)	0.035
Protein, gm	77.83 (1.20)	79.85 (1.15)	83.67 (1.68)	82.47 (2.10)	0.009
Laboratory data					
Blood cadmium, ug/L	0.27 (0.01)	0.32 (0.01)	0.40 (0.02)	0.40 (0.02)	< 0.001
Serum cotinine, ng/mL	11.42 (1.23)	22.91 (2.00)	36.13 (3.69)	39.03 (5.15)	< 0.001
Hemoglobin, g/dL	14.07 (0.05)	14.36 (0.06)	14.54 (0.05)	14.58 (0.07)	< 0.001
FBG, mg/dL	86.23 (0.49)	86.1 (0.29)	87.06 (0.43)	87.90 (0.41)	0.018
TC, mg/dL	163.98 (0.87)	161.76 (1.02)	160.58 (1.39)	159.64 (1.04)	0.001
Triglycerides, mg/dL	96.07 (2.41)	92.13 (1.55)	89.82 (1.88)	90.03 (1.68)	0.015
HDL-C, mg/dL	51.09 (0.32)	50.13 (0.36)	49.24 (0.46)	48.66 (0.48)	< 0.001
eGFR, mL/min per 1.73 m^2^	139.97 (0.73)	145.55 (0.8)	148.76 (0.92)	147.83 (0.76)	< 0.001
SUA, mg/dL	4.85 (0.04)	5.16 (0.04)	5.26 (0.04)	5.41 (0.06)	< 0.001
BUN, mg/dL	10.41 (0.13)	10.57 (0.13)	10.96 (0.13)	10.86 (0.18)	0.006
CRP, mg/dL	0.20 (0.01)	0.18 (0.01)	0.15 (0.01)	0.15 (0.02)	0.001

*Abbreviations*: *BLL* blood lead levels; *BMI* body mass index, *SBP* systolic blood pressure, *DBP* diastolic blood pressure, *TMFA* total monounsaturated fatty acids, *TPFA* total polyunsaturated fatty acids, *TSFA* total saturated fatty acids, *FBG* fasting blood glucose, *TC* total cholesterol, *HDL-C* high density lipoprotein cholesterol, *eGFR* estimated glomerular filtration rate, *SUA* serum uric acid, *BUN* blood urea nitrogen, *CRP* C-reactive protein

^*^Data are presented as weighted means, proportions and Se

^¶^The Physical Activity categories were based on the distribution of MET-minute levels for the present NHANES sample

^§^BMI was calculated as the body weight in kilograms divided by the square of the height in meters

^†^ Convert to BLL quartiles range: < 0.8 μg/dL, 0.8–1.2 μg/dL, 1.2–1.9 μg/dL, ≥ 1.9 μg/dL

For those subjects excluded from the analyses due to missing SUA or BLL values, their baseline characteristics were similar to their counterparts who were included in the analyses (Additional file 1: Table S1).

Table 2 showed the association of SUA with LnBLL using multivariate linear regression analyses. In the crude model, continuous LnBLL was positively correlated with SUA level (β = 0.29, 95%CI: 0.24–0.33, *P* <  0.001). After adjustment for different confounders, the positive association between LnBLL and SUA was still found in models 1–4. Similarly, in the fully adjusted model (model 3), for every 1 μg/dL increase in LnBLL, estimates from regression coefficients (β) indicated that the change in SUA was 0.13 mg/dL (95%CI: 0.09–0.17). We also converted LnBLL from a continuous variable to a categorical variable (quartiles). Compared to LnBLL < − 0.22 μg/dL (BLL <  0.80 μg/dL), there was a statistically significant higher SUA level for the participants in the second, third and highest LnBLL quartiles. *P* for trend in all of the models was significant and consistent with the *P* value when LnBLL was used as a continuous variable, suggesting the linear association between LnBLL and SUA. Table 3 presented the relative odds of having an elevated SUA. The results showed that LnBLL was independently and positively associated with elevated SUA in adolescents. In the fully adjusted model 3, compared to participants in the lowest LnBLL quartile, participants in the highest LnBLL quartile were associated with a 49% increased prevalence of elevated SUA. Also, *P* for trend in all models was significant.

**Table 2:** Association of SUA with LnBLL among 8303 12–19 year-old adolescents, NHANES 1999–2006

LnBLL, μg/dL	SUA, mg/dL, β (95%CI), *P* value				
	Crude model	Model 1	Model 2	Model 3	Model 4
Per 1 μg/dL increase	0.29 (0.24, 0.33), < 0.001	0.14 (0.11, 0.18), < 0.001	0.14 (0.10, 0.17), < 0.001	0.13 (0.09, 0.17), < 0.001	0.14 (0.10, 0.17), < 0.001
Quartiles					
Q1 (< −0.22)	Reference (0)	Reference (0)	Reference (0)	Reference (0)	Reference (0)
Q2 (−0.22–0.18)	0.23 (0.17, 0.30), < 0.001	0.13 (0.08, 0.19), < 0.001	0.11 (0.05, 0.16), < 0.001	0.10 (0.05, 0.16), < 0.001	0.11 (0.05, 0.16), < 0.001
Q3 (0.18–0.64)	0.31 (0.23, 0.38), < 0.001	0.13 (0.07, 0.19), < 0.001	0.11 (0.05, 0.17), < 0.001	0.10 (0.04, 0.16), 0.001	0.11 (0.05, 0.17), < 0.001
Q4 (≥ 0.64)	0.51 (0.43, 0.60), < 0.001	0.26 (0.19, 0.33), < 0.001	0.26 (0.19, 0.33), < 0.001	0.24 (0.17, 0.31), < 0.001	0.26 (0.19, 0.33), < 0.001
*P* for trend	< 0.001	< 0.001	< 0.001	< 0.001	< 0.001

Model 1was adjusted for sex, age, BMI, race, education status and physical activity

Model 2 was adjusted for all covariables in model 1 plus adjusted for SBP, DBP, blood cadmium, serum cotinine, hemoglobin, fasting blood glucose, total cholesterol, triglycerides, HDL-C, eGFR, blood urea nitrogen and C-reactive protein

Model 3 was adjusted for all covariables in model 2 plus adjusted for calcium intake, total monounsaturated fatty acids intake, total polyunsaturated fatty acids intake, total saturated fatty acids intake, total fat intake and total protein intake

Model 4 was adjusted for sex, age, BMI, race, hemoglobin, HDL-C and eGFR. The covariance was determined based on the matched odds ratio changed at least 10% when added to this model

*Abbreviations*: *BLL* blood lead levels, *SUA* serum uric acid, *CI* confidence interval

**Table 3:** Relative odds of having an elevated SUA levels among 8303 12–19 year-old adolescents, NHANES 1999–2006

LnBLL, μg/dL	Events (%)	Elevated SUA OR (95%CI), *P* value				
		Crude model	Model 1	Model 2	Model 3	Model 4
Per 1 μg/dL increase	2756 (33.2)	1.56 (1.45, 1.68), < 0.001	1.29 (1.18, 1.42), < 0.001	1.29 (1.17, 1.42), < 0.001	1.24 (1.11, 1.38), < 0.001	1.29 (1.17, 1.42), < 0.001
Quartiles						
Q1 (<−0.22)	666 (25.8)	Reference (1)	Reference (1)	Reference (1)	Reference (1)	Reference (1)
Q2 (− 0.22–0.18)	802 (32.8)	1.40 (1.24, 1.59), < 0.001	1.21 (1.04, 1.41), 0.015	1.13 (0.96, 1.32), 0.135	1.10 (0.93, 1.30), 0.267	1.13 (0.96, 1.32), 0.135
Q3 (0.18–0.64)	694 (36.2)	1.63 (1.44, 1.86), < 0.001	1.25 (1.07, 1.47), 0.007	1.18 (1.00, 1.39), 0.056	1.14 (0.95, 1.36), 0.157	1.18 (1.00, 1.39), 0.056
Q4 (≥ 0.64)	594 (43.9)	2.25 (1.96, 2.59), < 0.001	1.65 (1.38, 1.97), < 0.001	1.63 (1.35, 1.96), < 0.001	1.49 (1.22, 1.81), < 0.001	1.63 (1.35, 1.96), < 0.001
*P* for trend		< 0.001	< 0.001	< 0.001	< 0.001	< 0.001

Model 1was adjusted for sex, age, BMI, race, education status and physical activity

Model 2 was adjusted for all covariables in model 1 plus adjusted for SBP, DBP, blood cadmium, serum cotinine, hemoglobin, fasting blood glucose, total cholesterol, triglycerides, HDL-C, eGFR, blood urea nitrogen and C-reactive protein

Model 3 was adjusted for all covariables in model 2 plus adjusted for calcium intake, total monounsaturated fatty acids intake, total polyunsaturated fatty acids intake, total saturated fatty acids intake, total fat intake and total protein intake

Model 4 was adjusted for sex, age, BMI, race, hemoglobin, HDL-C and eGFR. The covariance was determined based on the matched odds ratio changed at least 10%

*Abbreviations*: *BLL* blood lead levels, *SUA* serum uric acid, *OR* odds ratio, *CI* confidence interval

We further explored the dose-response relation between BLL by a decile approach [≤0.5 (reference), 0.5–0.6, 0.6–0.7, 0.7–0.8, 0.8–0.9, 0.9–1.1, 1.1–1.4, 1.4–1.7, 1.7–2.3, and > 2.3 μg/dL] and SUA and elevated SUA (Additional file 1: Table S2 and Table S3). The overall pattern lent further support for a dose-response relation between BLL levels and SUA and elevated SUA (*P* for trend < 0.001).

**Fig. 1:**
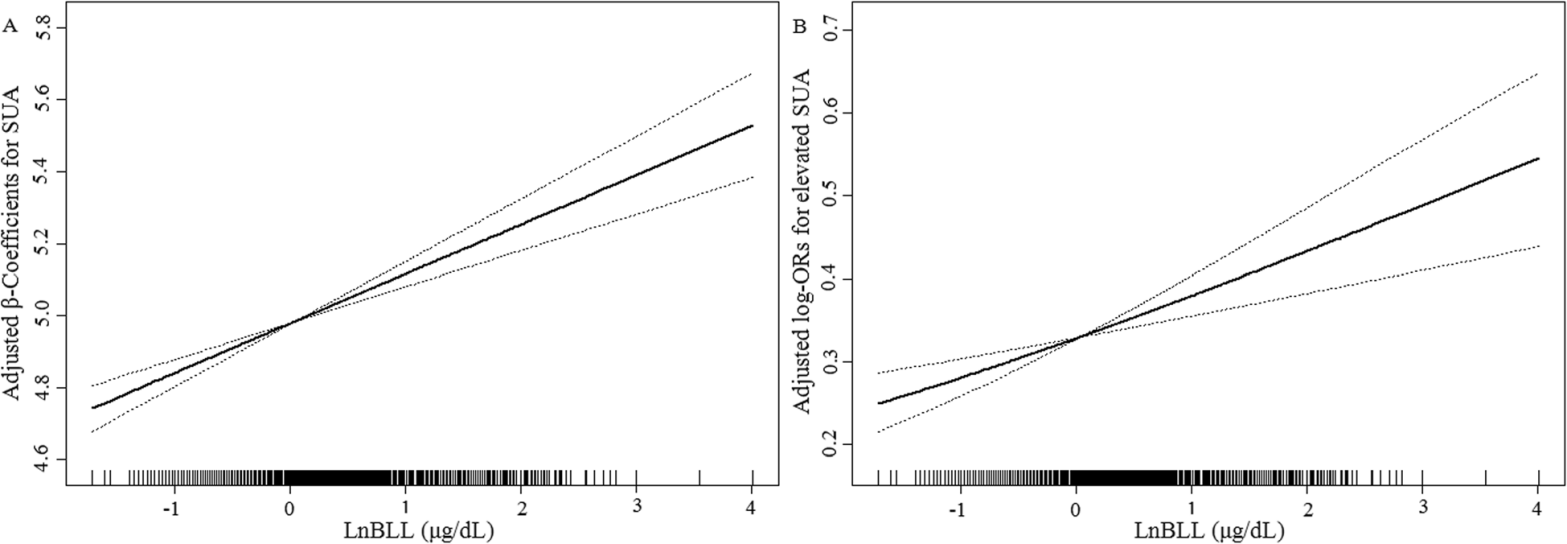
Dose–response relationship between LnBLL exposure and SUA^*^. **a** LnBLL and SUA; **b** LnBLL and elevated SUA. Abbreviations: BLL, blood lead levels; SUA, serum uric acid. ^*^Adjusted for sex, age, race, education status and physical activity; BMI, SBP, DBP, blood cadmium, serum cotinine, hemoglobin, fasting blood glucose, total cholesterol, triglycerides, HDL-C, eGFR, blood urea nitrogen, C-reactive protein, calcium intake, total monounsaturated fatty acids intake, total polyunsaturated fatty acids intake, total saturated fatty acids intake, total fat intake and total protein intake

Further analyses using restricted cubic spline confirmed the dose-relationship between LnBLL exposure and SUA (Fig. 1). Figure 1a and b showed that the associations of LnBLL with SUA and elevated SUA were linear.

**Fig. 2:**
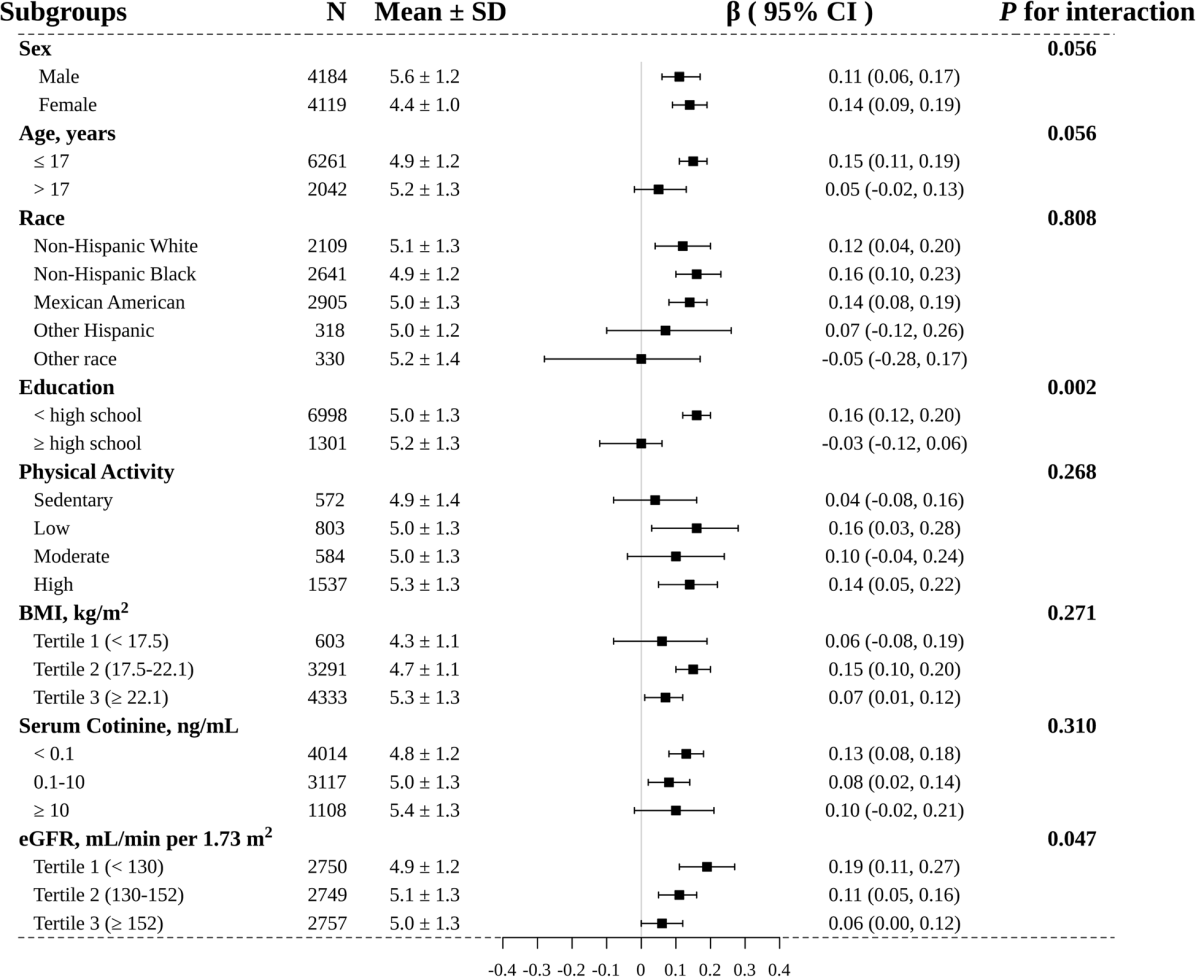
Subgroup analyses of the effect of LnBLL on SUA. Abbreviations: BLL, blood lead levels; SUA, serum uric acid; BMI, body mass index; eGFR, estimated glomerular filtration rate; CI, confidence interval. Adjusted for sex, age, race, education status and physical activity; BMI, SBP, DBP, blood cadmium, serum cotinine, hemoglobin, fasting blood glucose, total cholesterol, triglycerides, HDL-C, eGFR, blood urea nitrogen, C-reactive protein, calcium intake, total monounsaturated fatty acids intake, total polyunsaturated fatty acids intake, total saturated fatty acids intake, total fat intake and total protein intake, if not be stratified

In subgroup analysis, we further explored the role of other covariables on the association between LnBLL and SUA. As shown in Fig. 2, the subgroup analysis revealed a highly consistent pattern. Regardless of subgroup, LnBLL was positively associated with SUA. The effect of LnBLL on SUA was more significant at low education levels (< high school: β = 0.16, 95%CI: 0.12–0.20; ≥ high school: β = − 0.03, 95%CI: − 0.12-0.06, *P* for interaction = 0.002). Compared to subjects with eGFR ≥130 mL/min per 1.73 m^2^, those with eGFR < 130 mL/min per 1.73 m^2^ had a higher SUA level (β = 0.19, 95%CI, 0.11–0.27, *P* for interaction = 0.047) in subjects with high LnBLL. However, the association between LnBLL and SUA were consistent in the following subgroups: sex, age, race, physical activity, BMI and serum cotinine (*P* for interaction > 0.05 for all covariates).

## Discussion

Lead is a multiple-source pollutant well known for its adverse effects, and the main target organs of lead are the hematopoietic, nervous, and renal systems [29]. There are a number of similarities between the renal and vascular effects reported from low-level SUA and those from lead exposure. Tubulointerstitial fibrosis, a classic (although nonspecific) finding in lead exposure, has been observed in the uric acid model in the absence of the urate crystals that are commonly seen in this pathology at higher levels of hyperuricemia. Interestingly, in this cross-sectional study, we found that BLL was independently and positively associated with SUA level and elevated SUA in a representative sample of U.S. adolescents with relatively low BLL exposure. Also, our study confirmed a significant linear relationship between BLL and SUA level. Moreover, the subgroup analyses showed that stronger associations between BLL and SUA were detected in adolescent populations with lower levels of education and eGFR.

BLL has been found to be associated with SUA and hyperuricemia in multiple large epidemiologic studies in adults. Eswar Krishnan et al. [17]. used data from 6153 US adults aged 40 years or older and found that low level lead exposure (< 1.21 μmol/L) which was currently considered acceptable in adults was associated with increased prevalence of gout and hyperuricemia. Each doubling of BLL was associated with an unadjusted odds ratio of 1.74 (95%CI, 1.47 to 2.05) for gout and 1.25 (95%CI, 1.12 to 1.40) for hyperuricemia. Haijiang Dai et al. [12]. conducted a cross-sectional study of 2120 Chinese subjects aged ≥20 years and found that continuous lead exposure had an independent impact on SUA for both males and females, although this impact was more pronounced for females than for males. Moreover, the study also showed lead exposure was significantly associated with hyperuricemia for females but not for males.

However, several reports have also yielded some conflicting results. A longitudinal study conducted in Korean lead workers showed that BLL exposure was not associated with SUA [30]. A similar result was also reported by Omae et al. [31]. These conflicting results might be attributed to the differences in cohort characteristics, sample size, and adjustment of confounders. As we know, adults are more likely to have comorbidities, such as hypertension, chronic kidney disease (CKD), even smoking, which can make this relationship between BLL and SUA more complex. While in our study, we reported for the first time the relationship between BLL and SUA in adolescents, a population generally free from smoking, hypertension, diabetes mellitus or CKD. Therefore, they are an ideal population in which to examine this relationship. We found that continuous low level blood lead exposure was independently and positively associated with SUA. Further refinement in national goals for prevention, detection, and removal of lead from the environment should be pursued.

The mechanism driving this association is still unclear. However, several possible reasons could account for the association between BLL and SUA. BLL is an environmental pollutant that exhibits nephrotoxic activity, frequently evidenced by decreased GFR [3]. A review indicated that lead contributes to nephrotoxicity, even at BLL below 5 μg/dL^3^. BLL exposure could cause tubulointerstitial nephropathy, responsible for hyperuricemia and toxic effects on blood nucleo proteins that alter the metabolism of purine, thereby causing hyperuricemia [32]. The association between BLL and eGFR may be bidirectional [33]. In our study, we found that the effect of BLL on SUA was more pronounced in populations with lower eGFR. A possible reason for theses results is that lower eGFR may lead to an increase in SUA and elevated BLL by excretion disorders [34, 35]. Nevertheless, further studies are warranted to elucidate the mechanism underlying the role of BLL in kidney function. Furthermore, our subgroup analyses also showed that the effect of BLL on SUA was more pronounced in populations with a low education level. There was no association between BLL and SUA among adolescents with more than a high school level of education. The results were consistent in different age groups (the data was not shown in our paper). These findings suggest that life style, behavior or environmental conditions related to lower education has an indirect influence on the association between BLL exposure and SUA. Further research is needed to examine the relationship between education level, BLL and SUA.

Our study has some strengths. First, this study was the first report to explore the association between BLL and SUA in U.S. adolescents with relatively low BLL exposure; Second, we used a representative sample of the general adolescent population of the US from NHANES which applied rigorous quality controls to the procedures. Third, we adjusted for most potential confounders and effect modifiers. Fourth, we handled the target independent variable as both a continuous variable and as a categorical variable. Such an approach can reduce the contingency in the data analysis and enhance the robustness of results.

Some limitations of our study should be noted. First, the main limitations of this study was its observational nature. Second, as a cross-sectional design, it had less power to infer the causal association between BLL and SUA. Further prospective follow-up studies are needed to verify these findings. Third, further selection bias might have occurred because participants with missing BLL and SUA values were excluded. However, for the excluded population, their baseline characteristics were similar to their counterparts who were included in the analyses. Fourth, the BLL indicate recent exposure, but do not necessarily reflect the total bone lead burden which can provide a more valuable measure of internal dosage.

## Conclusion

In summary, continuous low level blood lead exposure was independently and positively associated with SUA in U.S. adolescents, and this impact was more pronounced in populations with lower levels of education and eGFR. The data suggest that there is no “safe” threshold level of exposure to lead. Further refinement in national goals for prevention, detection, and removal of lead from the environment should be pursued.

## Supplementary information

**Additional file 1: Table S1.** Characteristics of the included and excluded population. **Table S2.** Association of SUA with BLL levels-a dose-response analysis. **Table S3.** Association of elevated SUA with BLL levels-a dose-response analysis.

## Data Availability

The datasets are available on 10.5061/dryad.d5h62.

## Abbreviations

BLL: Blood lead levels
BMI: Body mass index
BUN: Blood urea nitrogen
CRP: C-reactive protein
DBP: Diastolic blood pressure
eGFR: estimated glomerular filtration rate
FBG: Fasting blood glucose
HDL-C: high density lipoprotein cholesterol
SBP: Systolic blood pressure
SUA: Serum uric acid
TC: Total cholesterol
TMFA: Total monounsaturated fatty acids
TPFA: Total polyunsaturated fatty acids
TSFA: Total saturated fatty acids
