# Supplementary material for: Association of low-level blood lead with serum uric acid in U.S. adolescents: a cross-sectional study
Source: Environ Health. 2019 Oct 16;18:86. doi: 10.1186/s12940-019-0524-0 (PMC6794798; doi:10.1186/s12940-019-0524-0)
Supplement: Supplementary file 1 — Additional file 1: Table S1. Characteristics of the included and excluded population. Table S2. Association of SUA with BLL levels-a dose-response analysis. Table S3. Association of elevated SUA with BLL levels-a dose-response analysis. [file 12940_2019_524_MOESM1_ESM.doc]

Additional file 1: Table S1. Characteristics of the included and excluded population

| Variable | Included | Excluded | *P* value |
| --- | --- | --- | --- |
| N | 8303 | 1190 |  |
| Male, % | 4184 (50.4) | 571 (48.0) | 0.120 |
| age, years | 15.5 ± 2.3 | 15.4 ± 2.3 | 0.056 |
| BMI, kg/m^2^ | 24.0 ± 6.0 | 23.7 ± 6.6 | 0.262 |
| SBP, mm Hg | 110.0 ± 10.1 | 109.6 ± 10.3 | 0.333 |
| DBP, mm Hg | 60.7 ± 11.7 | 60.7 ± 12.8 | 0.956 |
| **Race, %** |  |  | 0.008 |
| Non-Hispanic White | 2109 (25.4) | 329 (27.6) |  |
| Non-Hispanic Black | 2641 (31.8) | 376 (31.6) |  |
| Mexican American | 2905 (35.0) | 368 (30.9) |  |
| Other Hispanic | 318 (3.8) | 51 (4.3) |  |
| Other race | 330 (4.0) | 66 (5.5) |  |
| **Education, %** |  |  | 0.822 |
| < high school | 6998 (84.3) | 1006 (84.8) |  |
| High school | 678 (8.2) | 97 (8.2) |  |
| > high school | 623 (7.5) | 83 (7.0) |  |
| **Physical Activity, %** |  |  | 0.302 |
| Sedentary | 572 (16.4) | 92 (19.9) |  |
| Low | 803 (23.0) | 100 (21.6) |  |
| Moderate | 584 (16.7) | 75 (16.2) |  |
| High | 1537 (44.0) | 196 (42.3) |  |
| **Dietary** |  |  |  |
| Calcium, mg | 923.3 ± 602.4 | 878.3 ± 560.7 | 0.061 |
| TMFA, gm | 31.3 ± 17.1 | 29.8 ± 16.8 | 0.021 |
| TPFA, gm | 16.4 ± 10.5 | 15.7 ± 10.6 | 0.083 |
| TSF, gm | 28.4 ± 15.9 | 26.7 ± 15.0 | 0.004 |
| Total fat, gm | 82.8 ± 43.6 | 78.6 ± 42.6 | 0.011 |
| Protein, gm | 78.1 ± 40.2 | 74.5 ± 39.1 | 0.019 |
| **Laboratory data** |  |  |  |
| BLL, ug/dL | 1.3 ± 1.3 | 1.3 ± 1.2 | 0.718 |
| Blood cadmium, ug/L | 0.3 ± 0.3 | 0.3 ± 0.2 | 0.091 |
| Serum cotinine, ng/mL | 17.5 ± 58.5 | 12.6 ± 41.3 | 0.577 |
| Hemoglobin, g/dL | 14.1 ± 1.4 | 14.0 ± 1.5 | 0.422 |
| FBG, mg/dL | 86.6 ± 14.8 | 85.7 ± 11.2 | 0.876 |
| TC, mg/dL | 162.4 ± 31.8 | 175.8 ± 38.2 | 0.045 |
| Triglycerides, mg/dL | 88.0 ± 61.4 | 103.4 ± 79.3 | 0.507 |
| HDL-C, mg/dL | 50.9 ± 12.1 | 50.9 ± 10.0 | 0.999 |
| eGFR, mL/min per 1.73 m^2^ | 144.1 ± 24.2 | 155.5 ± 29.4 | 0.213 |
| SUA, mg/dL | 5.0 ± 1.3 | 5.2 ± 0.7 | 0.637 |
| BUN, mg/dL | 10.3 ± 3.3 | 9.1 ± 2.0 | 0.351 |
| CRP, mg/dL | 0.2 ± 0.5 | 0.2 ± 0.5 | 0.421 |

Abbreviations: BMI, body mass index; SBP, systolic blood pressure; DBP, diastolic blood pressure; TMFA, total monounsaturated fatty acids; TPFA, total polyunsaturated fatty acids; TSFA, total saturated fatty acids; BLL, blood lead levels; FBG, fasting blood glucose; TC, total cholesterol; HDL-C, high density lipoprotein cholesterol; eGFR, estimated glomerular filtration rate; SUA, serum uric acid; BUN, blood urea nitrogen; CRP, C-reactive protein.

Table S2. Association of SUA with BLL levels-a dose-response analysis

| **BLL, μg/dL** | **SUA, mg/dL, β (95%CI), *P* value** | | | |
| --- | --- | --- | --- | --- |
|  | Crude model | Model 1 | Model 2 | Model 3 |
| ≤0.5 | Reference (0) | Reference (0) | Reference (0) | Reference (0) |
| 0.5-0.6 | 0.10 (-0.02, 0.22) | 0.07 (-0.02, 0.17) | 0.08 (-0.02, 0.17) | 0.08 (-0.02, 0.17) |
| 0.6-0.7 | 0.22 (0.10, 0.34) | 0.14 (0.05, 0.23) | 0.12 (0.03, 0.21) | 0.12 (0.03, 0.21) |
| 0.7-0.8 | 0.22 (0.10, 0.34) | 0.12 (0.03, 0.22) | 0.10 (0.01, 0.19) | 0.10 (0.01, 0.19) |
| 0.8-0.9 | 0.33 (0.21, 0.45) | 0.16 (0.07, 0.25) | 0.15 (0.06, 0.24) | 0.15 (0.06, 0.24) |
| 0.9-1.1 | 0.40 (0.30, 0.51) | 0.23 (0.14, 0.31) | 0.19 (0.11, 0.27) | 0.19 (0.11, 0.27) |
| 1.1-1.4 | 0.40 (0.30, 0.51) | 0.18 (0.10, 0.27) | 0.15 (0.07, 0.23) | 0.15 (0.07, 0.23) |
| 1.4-1.7 | 0.41 (0.29, 0.53) | 0.21 (0.11, 0.31) | 0.16 (0.06, 0.26) | 0.16 (0.06, 0.25) |
| 1.7-2.3 | 0.58 (0.47, 0.70) | 0.29 (0.19, 0.39) | 0.25 (0.15, 0.34) | 0.25 (0.15, 0.34) |
| >2.3 | 0.61 (0.50, 0.73) | 0.32 (0.23, 0.42) | 0.27 (0.17, 0.36) | 0.26 (0.17, 0.36) |
| *P* for trend | <0.001 | <0.001 | <0.001 | <0.001 |

Model 1 was adjusted for sex, age, BMI, race, education status and physical activity;

Model 2 was adjusted for all covariables in model 1 plus adjusted for SBP, DBP, blood cadmium, serum cotinine, hemoglobin, fasting blood glucose, total cholesterol, triglycerides, HDL-C, eGFR, blood urea nitrogen and C-reactive protein;

Model 3 was adjusted for all covariables in model 2 plus adjusted for calcium intake, total monounsaturated fatty acids intake, total polyunsaturated fatty acids intake, total saturated fatty acids intake, total fat intake and total protein intake;

Abbreviations: BLL, blood lead levels; SUA, serum uric acid; CI, confidence interval.

Table S3. Association of elevated SUA with BLL levels-a dose-response analysis

| **BLL, μg/dL** | **SUA, mg/dL, OR (95%CI), *P* value** | | | |
| --- | --- | --- | --- | --- |
|  | Crude model | Model 1 | Model 2 | Model 3 |
| ≤0.5 | Reference (1) | Reference (1) | Reference (1) | Reference (1) |
| 0.5-0.6 | 1.23 (0.98, 1.55) | 1.23 (0.93, 1.63) | 1.25 (0.94, 1.67) | 1.24 (0.93, 1.66) |
| 0.6-0.7 | 1.40 (1.12, 1.74) | 1.30 (0.99, 1.70) | 1.26 (0.96, 1.67) | 1.26 (0.95, 1.67) |
| 0.7-0.8 | 1.57 (1.27, 1.95) | 1.45 (1.11, 1.88) | 1.34 (1.02, 1.76) | 1.33 (1.01, 1.75) |
| 0.8-0.9 | 1.50 (1.20, 1.86 | 1.36 (1.12, 1.45) | 1.35 (1.11, 1.49) | 1.34 (1.10, 1.49) |
| 0.9-1.1 | 1.84 (1.52, 2.23) | 1.50 (1.18, 1.90) | 1.37 (1.06, 1.75) | 1.36 (1.06, 1.74) |
| 1.1-1.4 | 1.97 (1.63, 2.39) | 1.66 (1.15, 1.85) | 1.66 (1.06, 1.75) | 1.57 (1.06, 1.75) |
| 1.4-1.7 | 1.83 (1.47, 2.27) | 1.56 (1.01, 1.73) | 1.46 (1.01, 1.55) | 1.45 (1.00, 1.53) |
| 1.7-2.3 | 2.66 (2.17, 3.27) | 1.90 (1.47, 2.46) | 1.73 (1.32, 2.27) | 1.74 (1.32, 2.28) |
| >2.3 | 2.69 (2.20, 3.29) | 1.90 (1.47, 2.45) | 1.65 (1.26, 2.17) | 1.65 (1.26, 2.17) |
| *P* for trend | <0.001 | <0.001 | <0.001 | <0.001 |

Model 1 was adjusted for sex, age, BMI, race, education status and physical activity;

Model 2 was adjusted for all covariables in model 1 plus adjusted for SBP, DBP, blood cadmium, serum cotinine, hemoglobin, fasting blood glucose, total cholesterol, triglycerides, HDL-C, eGFR, blood urea nitrogen and C-reactive protein;

Model 3 was adjusted for all covariables in model 2 plus adjusted for calcium intake, total monounsaturated fatty acids intake, total polyunsaturated fatty acids intake, total saturated fatty acids intake, total fat intake and total protein intake;

Abbreviations: BLL, blood lead levels; SUA, serum uric acid; OR, odds ratio; CI, confidence interval.
